# Immunocyte lipid metabolic reprogramming: a novel pathway for targeted intervention in autoimmune diseases

**DOI:** 10.3389/fimmu.2025.1713148

**Published:** 2025-11-06

**Authors:** Yanyu Cui, Zhendong Feng, Qihan Zhao, Haoran Dai, Yang Zheng, Hongliang Rui, Baoli Liu

**Affiliations:** 1 Beijing Hospital of Traditional Chinese Medicine, Capital Medical University, Beijing, China; 2 Shunyi Branch, Beijing Traditional Chinese Medicine Hospital, Beijing, China; 3 Beijing Research Institute of Chinese Medicine, Beijing University of Chinese Medicine, Beijing, China

**Keywords:** lipid metabolism, immunometabolism, autoimmune diseases, T cells, B cells, macrophages, targeted therapy

## Abstract

Lipids orchestrate immune signaling beyond structure and energy. In autoimmune diseases (ADs), immune cells rewire fatty-acid and cholesterol pathways under microenvironmental pressures, creating pharmacologically actionable dependencies. This metabolic dysregulation is not merely a passive consequence of immune activation but is a key driver of disease progression. This review synthesizes evidence from human and preclinical studies to systematically outline the core regulatory networks of lipid metabolism. It further dissects the role of lipid metabolism in reshaping the functions of T cells, B cells, macrophages, and dendritic cells, and delineates its organ-specific dysregulation in various ADs (e.g., synovium, skin, central nervous system, gut). Rather than blanket immunosuppression, we propose “immune-metabolic normalization”: titrating hyperactive nodes to physiological set-points while preserving host defense. We prioritize targets with high translational potential and evaluate corresponding targeted strategies, including drug repurposing, novel agents in clinical development, and innovative interventional concepts. Our work aims to bridge descriptive immunometabolic research with verifiable, patient-centered interventions, laying the groundwork for precision medicine in autoimmune diseases.

## 1 Introduction

Autoimmune diseases (ADs) are chronic inflammatory conditions characterized by a loss of immune tolerance to self-antigens, leading to immune-mediated attack on the body’s own tissues and organs (1). Affecting approximately 3-5% of the global population with rising incidence, ADs pose a significant public health challenge (2). Current clinical management primarily relies on glucocorticoids, broad-spectrum immunosuppressants, and biologics (3). However, these therapies often lack specificity; while suppressing aberrant immune responses, they concurrently impair normal host defense mechanisms, increasing the risk of infections and malignancies. Furthermore, many patients experience treatment resistance, disease relapse, or drug intolerance, highlighting the limitations of current strategies and underscoring the urgent need for more precise, safe, and durable interventions (3).

The emergence of the immunometabolism field has provided a revolutionary perspective for understanding and treating immune-mediated diseases. This discipline has revealed that cellular metabolism is not merely a passive process supplying energy post-activation (4) but is a decisive factor actively dictating immune cell fate and function (5, 6). Immune cells undergo precise metabolic reprogramming during different stages—quiescence, activation, differentiation, and memory formation—to meet specific bioenergetic and biosynthetic demands. For instance, effector T cells rely on glycolysis (7), whereas regulatory T cells (Tregs) and memory T cells prefer oxidative phosphorylation (OXPHOS) (8). This tight coupling between metabolic signatures and cellular functions implies that metabolic pathways themselves can be targeted to modulate immune responses. By intervening at metabolic vulnerabilities specific to pathogenic immune cell subsets, it may be possible to selectively suppress deleterious immunity while sparing protective functions, enabling more precise immunomodulation.

Within the broad landscape of immunometabolism, lipid metabolism is moving from the background to the forefront. Traditionally viewed primarily as structural membrane components and energy stores, lipids are now recognized for their complex and central regulatory functions. They act as diverse signaling molecules (e.g., sphingosine-1-phosphate [S1P], specialized pro-resolving mediators [SPMs]) (9, 10), directly regulating cell survival, migration, and activation. They form the basis of key signaling platforms—lipid rafts—where minor compositional changes can influence the signaling efficiency of immune receptors like the T-cell receptor (TCR) and B-cell receptor (BCR) (11–13). Furthermore, lipid metabolites serve as substrates for post-translational modifications (e.g., palmitoylation) or ligands for nuclear receptors (e.g., Peroxisome Proliferator-Activated Receptors [PPARs], Liver X Receptors [LXRs]), thereby reshaping the gene expression profiles of immune cells at the transcriptional level (11, 14). Metaflammation represents a core link connecting metabolic dysregulation and autoimmunity (15). Growing evidence strongly indicates that lipid metabolic dysregulation is a central pathological feature of numerous ADs, including systemic lupus erythematosus (SLE), rheumatoid arthritis (RA), multiple sclerosis (MS), inflammatory bowel disease (IBD), and psoriasis (16). Metabolically derived harmful products can activate immune cells, while inflammatory cytokines released during immune responses exacerbate metabolic imbalance, creating a vicious cycle that drives AD pathogenesis (17).

This review aims to systematically elaborate on the role of immunocyte lipid metabolic reprogramming in ADs and its potential as a therapeutic target. We will first overview the fundamental biochemical processes, key regulatory networks, and organellar basis of lipid metabolism. Subsequently, we will detail how lipid metabolism finely regulates the differentiation and function of key immune cells, including T cells, B cells, and antigen-presenting cells (APCs). Then, we will delve into the specific manifestations and pathogenic mechanisms of lipid metabolic dysregulation in five representative ADs: SLE, RA, MS, IBD, and psoriasis. Finally, we will comprehensively evaluate therapeutic strategies targeting lipid metabolism. These strategies encompass drug repurposing, novel agents under clinical development, promising preclinical targets, and non-pharmacological interventions. This work is intended to provide a theoretical basis and direction for future research and clinical translation in this field.

## 2 Dual roles of lipid metabolism in immune regulation

Lipids are chemically and functionally diverse molecules, including fatty acids (FAs), triglycerides, cholesterol, and sphingolipids (18, 19). Their functions extend far beyond energy storage and biomembrane constitution (20–42; **Supplementary Table 1**). FAs are not only energy sources and precursors for complex lipids but also give rise to potent inflammatory mediators like prostaglandins (PGs) and leukotrienes (10). Sphingolipid metabolites, such as ceramide and S1P, are important second messengers regulating apoptosis, proliferation, and migration (43). Cholesterol is crucial for maintaining membrane fluidity and integrity and serves as a precursor for steroid hormones and bile acids (44). The dynamic balance among these molecules collectively determines cellular structural integrity, signaling efficiency, and metabolic status. This section outlines the core components of lipid metabolism to provide a foundation for understanding its cell-specific reprogramming in immune cells.

### 2.1 Coordination of key metabolic pathways and organelles

**Figure 1:**
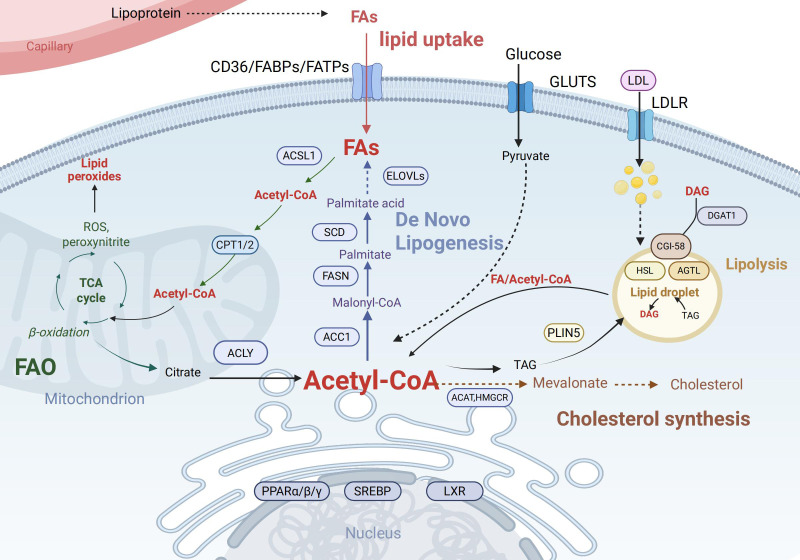
Key pathways of intracellular lipid metabolism. Cellular uptake of fatty acids (FAs) occurs via CD36, FABPs, and FATPs, while LDL enters through LDLR. Fas are activated to acyl-CoA by ACSL1 and can either undergo mitochondrial import via CPT1/2 for β-oxidation (producing acetyl-CoA, ATP, ROS, and citrate) or serve as substrates for *de novo* lipogenesis involving ELOVLs, SCD, and FASN to generate palmitate and triacylglycerol (TAG). Glucose-derived pyruvate also contributes to Acetyl-CoA production. TAG stored in lipid droplets is hydrolyzed by ATGL and HSL under PLIN5 regulation, releasing Diacylglycerol (DAG) and acyl-CoA, which may be re-esterified by DGAT1. Acetyl-CoA is also utilized for cholesterol synthesis via the mevalonate pathway (catalyzed by HMGCR). Key transcription factors including PPARs, Sterol Regulatory Element-Binding Proteins (SREBPs), and Liver X Receptors (LXRs) coordinate these processes by regulating lipid metabolic genes. Created in BioRender. Yu, Y (2025). https://BioRender.com/havvjba Abbreviations: CD36,Cluster of Differentiation 36; FABPs, Fatty Acid-Binding Proteins; FATPs, Fatty Acid Transport Proteins; LDL, Low-Density Lipoprotein; LDLR, Low-Density Lipoprotein Receptor; ACSL1, Acyl-CoA Synthetase Long-Chain Family Member 1; CPT1/2, Carnitine Palmitoyltransferase 1 and 2; ROS, Reactive Oxygen Species; ELOVLs, Elongation of Very Long Chain Fatty Acids Proteins; SCD, Stearoyl-CoA Desaturase; FASN, Fatty Acid Synthase; TAG, Triacylglycerol; ATGL, Adipose Triglyceride Lipase; HSL, Hormone-Sensitive Lipase; PLIN5, Perilipin 5; DAG, Diacylglycerol; DGAT1, Diacylglycerol O-Acyltransferase 1;HMGCR, 3-Hydroxy-3-Methylglutaryl-CoA Reductase; PPARs, Peroxisome Proliferator-Activated Receptors.

The fate and function of immune cells depend on the reprogramming of key lipid metabolic pathways, particularly the balance between *de novo* lipogenesis (DNL) and fatty acid β-oxidation (FAO) (**Figure 1**).

Lipid metabolism depends on a highly coordinated network of organelles. DNL, which initiates from acetyl-CoA, occurs primarily in the endoplasmic reticulum (ER) (45, 46), whereas FAO takes place in mitochondria. Mitochondrial fitness—encompassing membrane potential, respiratory capacity, and dynamics (fusion and fission)—is critical for immune cell function (47, 48). Mitochondria also establish close contact with the endoplasmic reticulum (ER) via structures known as mitochondria-associated membranes (MAMs). These MAMs play a key role in multiple cellular processes: they mediate lipid transport, regulate calcium signaling, and help maintain cellular homeostasis (49). Furthermore, lipid droplets (LDs) store neutral lipids to buffer lipotoxicity and supply energy via lipolysis (50, 51). LDs also function as docking sites for signaling proteins and serve as platforms for the synthesis of inflammatory lipid mediators such as eicosanoids, thereby directly linking lipid storage to inflammatory responses (52, 53).

Different immune cells exhibit distinct preferences for metabolic pathways depending on their functional status. DNL supplies membrane constituents and modulates signaling in rapidly proliferating activated immune cells (54). In contrast, FAO serves as a crucial energy source required for the maintenance and survival of long-lived cells such as memory T cells and Tregs, thereby supporting their persistence and capacity for rapid responses (55). In these cells, fully functional mitochondria are indispensable (56, 57). FAO exerts diverse roles across immune cell types, and metabolic preferences evolve during cellular differentiation and in response to varying physiological contexts (55).

Cholesterol homeostasis is maintained through a balance of synthesis, uptake (via LDLR), and efflux (via ABCA1/ABCG1) (58). Together with sphingolipids, cholesterol forms specialized plasma membrane microdomains known as lipid rafts. These rafts act as signaling platforms that enrich the T-cell receptor (TCR), co-stimulatory molecules (e.g., CD28), and downstream signaling proteins (e.g., Lck). Upon antigen stimulation, lipid rafts facilitate the aggregation of these molecules, effectively initiating and amplifying TCR signaling (59, 60). Increased membrane cholesterol content lowers the activation threshold of T cells, a key mechanism underlying T cell hyperactivation in SLE patients (61, 62).

Under pathological conditions such as autoimmune disease, this precise metabolic coordination becomes disrupted. When immune cells encounter high protein synthesis demands (e.g., antibody secretion in plasma cells) or experience lipid imbalance, endoplasmic reticulum (ER) stress is triggered, activating the unfolded protein response (UPR) (63). Although the UPR attempts to mitigate stress by upregulating lipid synthesis genes to expand ER membrane capacity, persistent ER stress disrupts lipid metabolism and can trigger inflammatory or apoptotic signaling (64). In activated immune cells such as macrophages and neutrophils, the number and size of LDs increase significantly, representing a hallmark feature of immunometabolic reprogramming (65).

### 2.2 The lipid regulatory network modulates immune functions

A sophisticated molecular network within cells senses lipid and energy status to adjust metabolic pathways and thereby regulate immune function. Key sensory and signaling pathways include:

#### 2.2.1 Sterol regulatory element-binding proteins

SREBPs are master transcription factors regulated by cellular sterol levels (66). SREBP-2 primarily activates genes in the cholesterol synthesis pathway (e.g., HMG-CoA reductase [HMGCR], LDLR), while SREBP-1c mainly regulates genes involved in FA and triglyceride synthesis (e.g., acetyl-CoA carboxylase [ACC], fatty acid synthase [FASN]) (67). Studies have shown that the regulation of lipid synthesis can directly drive the functions of various effector immune cells. For instance, the SREBP signaling pathway in B cells is essential for antibody responses, as well as for the formation of germinal centers, memory B cells, and bone marrow plasma cells (68). In DCs, lipid metabolism regulates their antigen presentation and maturation processes (69).

#### 2.2.2 Peroxisome proliferator-activated receptors

PPARs are a family of nuclear receptors (PPARα, PPARβ/δ, PPARγ) acting as intracellular lipid sensors (70). PPARα and PPARβ/δ promote FAO and energy expenditure, whereas PPARγ drives lipogenesis and storage (71). PPARs play pivotal roles in integrating lipid metabolism with inflammation; for instance, PPARγ exerts potent anti-inflammatory effects and is a key determinant in the fate of Tregs, memory T cells, and M2 macrophages (72, 73). However, it is crucial to note this: PPARγ’s effects on immune cells are highly context-dependent (74). Additionally, they can vary substantially across species (75).

#### 2.2.3 Liver X receptors

LXRs (LXRα and LXRβ) are nuclear receptors activated by oxysterols, sensing cholesterol levels (76). They promote cholesterol efflux by upregulating ABCA1/G1 to prevent cellular cholesterol overload. Additionally, LXRs possess significant anti-inflammatory functions, inhibiting the expression of inflammatory genes in macrophages (77) and modulating T cell and dendritic cell (DC) function, relevant to ADs (78–80).

#### 2.2.4 AMPK/mTOR axis

AMP-activated protein kinase (AMPK) and the mechanistic target of rapamycin complex 1 (mTORC1) are central kinases sensing cellular energy and nutrient status, exerting antagonistic yet coordinated effects on lipid metabolism (73). AMPK, an energy sensor, activates under low energy conditions (high AMP/ATP ratio). Activated AMPK phosphorylates and inhibits synthases like ACC. This action shuts down energy-consuming anabolic processes while promoting energy-producing catabolism, thereby reprogramming cellular energy metabolism to support cell function. In regulatory T cells (Tregs), this metabolic reprogramming combines with AMPK-mediated Foxp3 phosphorylation and stabilization. Together, they maintain the suppressive capacity of Tregs. Defects in this process can lead to autoimmune liver disease (81). mTORC1, a nutrient sensor, activates when signals like amino acids, glucose, and growth factors are abundant. Activated mTORC1 promotes anabolic processes necessary for growth and proliferation, including protein synthesis and lipid synthesis (partly via SREBP activation) (82). The balance of the AMPK/mTOR axis determines whether a cell is in an anabolic or catabolic mode, crucially influencing immune cell fate (83).

Cellular lipid metabolic status is determined not by a single pathway but by the interconnected network of these pathways at the organellar, metabolic, and signaling levels (84) (**Supplementary Figure S1**). For example, LXRs can induce SREBP-1c expression, forming a feed-forward loop connecting cholesterol clearance to FA synthesis, facilitating the esterification and storage of free cholesterol as cholesterol esters in LDs, which is vital for lipid homeostasis but can contribute to pathology when dysregulated (85). This networked regulation provides metabolic plasticity but also means dysfunction at any node can trigger cascades leading to complex metabolic disturbances. In ADs, persistent inflammatory signals and altered nutrient environments impact multiple nodes simultaneously, disrupting homeostasis and driving pathological metabolic reprogramming. We will now explore how these principles are implemented across different immune cell subsets. This metabolic programming ultimately shapes their unique immune functions.

## 3 Lipid metabolic preferences and functional remodeling in key immune cells

**Figure 2:**
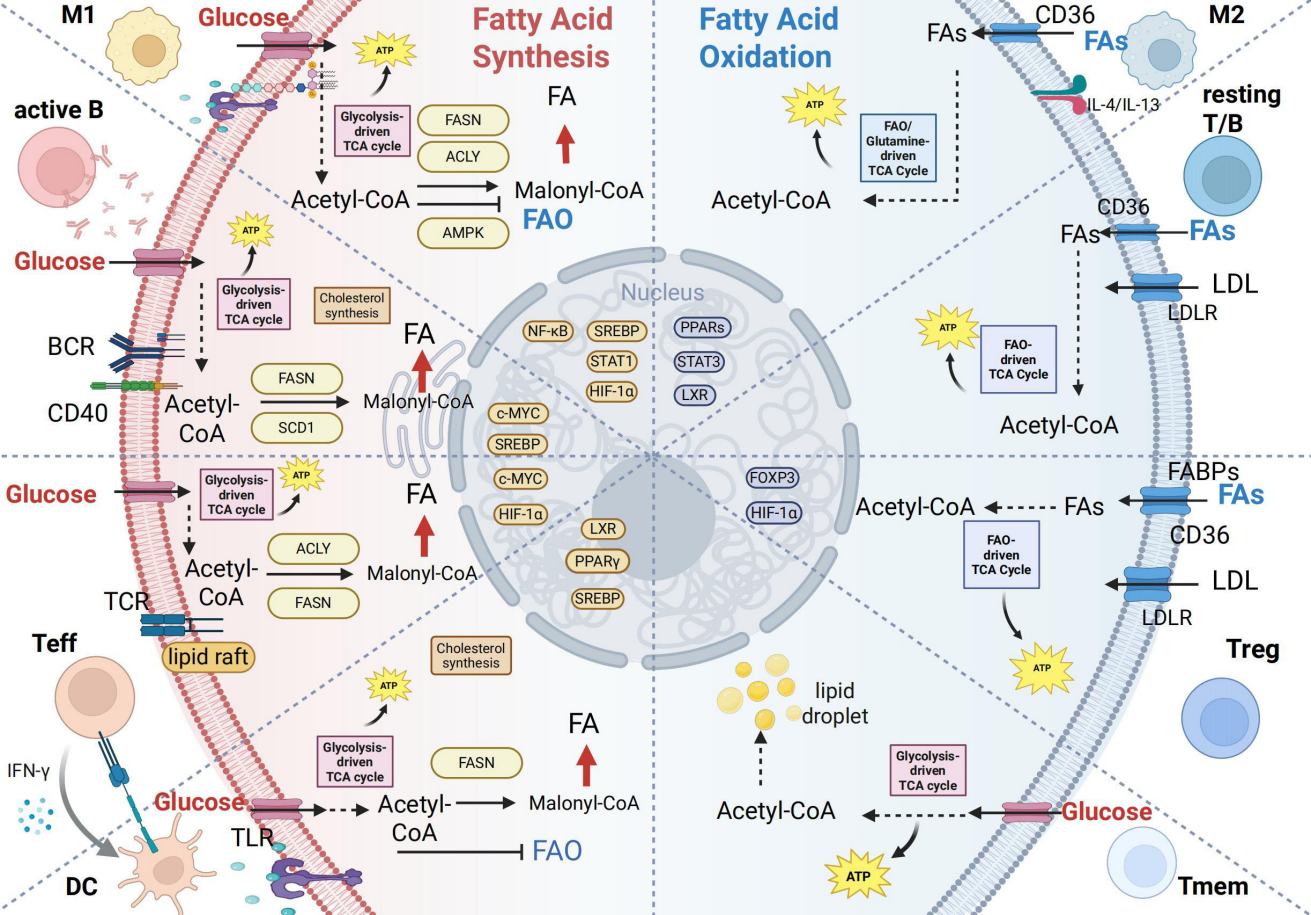
Lipid metabolic reprogramming in immune cell activation and differentiation. This figure summarizes lipid metabolic patterns across immune cell subsets, emphasizing the shift between fatty acid synthesis (FAS) and fatty acid oxidation (FAO) to meet functional demands. Left (Lipogenic phenotype – proliferating/effector cells): Rely on *de novo* lipogenesis (DNL) for membrane biogenesis and rapid growth.M1 macrophages: LPS/TLR4-induced HIF-1α enhances ACLY/FASN, promoting lipid synthesis and inflammation. Activated B cells: BCR/CD40 signaling activates SREBP/c-MYC, upregulating FASN/SCD1 to support antibody production. Effector T cells: TCR/mTOR-driven DNL facilitates proliferation and cytokine secretion. Dendritic cells: Glycolysis and DNL support antigen presentation structures. Right (FAO phenotype – regulatory/memory cells): Depend on fatty acid oxidation for long-term survival and regulatory functions.M2 macrophages: IL-4/IL-13 activate PPAR-γ/LXR, promoting FAO and anti-inflammatory activity. Resting T/B cells: Use exogenous lipids and FAO to maintain quiescence. Tregs: FAO-derived metabolites sustain Foxp3 and suppressive function. Memory cells: Rely on CD36 and FAO for rapid recall capacity. Transcriptional regulation: Key sensors (SREBP-1c, PPARs, HIF-1α/mTOR) integrate nutrient and immune signals to dynamically regulate metabolic genes, thereby coordinating immune cell function. Created in BioRender. yu, Y (2025). https://BioRender.com/yi5u4zy“.

Building upon the foundational framework of lipid metabolism, we have observed that immune cells exhibit functionally specific lipid metabolic preferences (**Figure 2**), which directly shape the intensity and type of immune response (55). This section will provide an in-depth analysis of how key immune cells—such as T cells, B cells, macrophages, and dendritic cells—leverage distinct lipid metabolic patterns to support their activation, differentiation, and functional execution (**Table 1**). Furthermore, it aims to elucidate the intrinsic link between metabolic reprogramming and the remodeling of immune functions.

**Table 1:** Lipid metabolic features of major immune cell subsets.

Cell type	Major metabolic pathway(s)	Key metabolic regulator(s)	Key differentiation/activation signal(s)	Immune function	Association with autoimmune diseases	References
Th17 cells	FAS	PI3K/Akt/mTORC1	STAT3	Produces IL-17/22, drives tissue inflammation	Linked to RA (synovial inflammation), MS (CNS inflammation), IBD (intestinal inflammation), and psoriasis (keratinocyte hyperproliferation).	(229, 230)
Treg cells	FAO	AMPK/PPARα/β	TGF-β/Smad3	Maintains immune tolerance, suppresses effector T cells	Frequently impaired in SLE, IBD, RA	(231, 232)
Memory T cells	FAO	AMPK/PPARδ	IL-7/IL-15	Supports long-term survival and rapid recall response	Contribute to MS relapses (central memory T cells), sustain chronic inflammation in RA (synovial memory T cells), and cooperate in pathogenesis in SLE (alongside memory B cells).	(148, 149, 233)
Plasma cells	FAS/cholesterol synthesis	SREBP-1c/mTORC1	BLIMP1	Large-scale antibody synthesis and secretion	Key contributors through autoantibody production: anti-nuclear/anti-dsDNA antibodies in SLE, anti-CCP antibodies in RA, and anti-SSA/Ro antibodies in Sjögren’s syndrome.	(233)
M1 macrophages	Glycolysis/FAS	HIF-1α	NF-κB/TLR4/MyD88	Releases pro-inflammatory cytokines, enhances bactericidal activity	Enriched in RA synovium (promoting joint destruction), promote lupus nephritis in SLE kidneys, and enhance keratinocyte proliferation in psoriasis.	(140, 234)
M2 macrophages	FAO	PPARγ	IL-4/STAT6 IL-10/STAT3	Tissue repair, secretes anti-inflammatory factors	Contribute to disease through loss of function: deficient intestinal barrier repair in IBD, impaired remyelination in MS, and weakened anti-inflammatory activity in RA.	(235)
Activated DC cells	Glycolysis/FAS	mTORC1/HIF-1α	TLR2/4-TRIF	Expresses co-stimulatory molecules, initiates T cell response	Critical in initiating localized autoimmunity: activate Th17 cells and promote CNS infiltration in MS, stimulate pathogenic T cells in psoriatic skin, and disrupt tolerance in IBD via excessive immune activation.	(136, 236, 237)

### 3.1 T cells

As central players in adaptive immunity, T cell differentiation and function are tightly controlled by their metabolic state. Lipid metabolism acts as a checkpoint determining T cell fate (113, 114).

#### 3.1.1 CD4+T cells

##### 3.1.1.1Metabolic regulation of Th17/Treg balance

Upon antigen activation, naïve T cells rapidly initiate metabolic reprogramming, shifting to aerobic glycolysis to support rapid clonal expansion. Their eventual differentiation into different effector subsets is closely linked to lipid metabolic pathway choices. Pathogenic Th17 cells heavily rely on the DNL/fatty acid synthesis (FAS) pathway (involving enzymes like ATP-citrate lyase [ACLY], ACC, FASN) to convert glucose-derived carbons into FAs (115). Inhibiting ACC1 or FASN pharmacologically (e.g., with TOFA or soraphen A) or genetically can effectively block Th17 differentiation and promote Treg generation, ameliorating disease in autoimmune models (8, 115). Conversely, immunosuppressive Treg cells depend on FAO to maintain their stability and function (116, 117). Early studies using the carnitine palmitoyltransferase 1a(CPT1a) inhibitor Etomoxir suggested that FAO is crucial for Treg cell function. This interpretation, however, was complicated by subsequent findings that questioned Etomoxir’s specificity and indicated CPT1a-independent effects (118). More definitive support comes from genetic studies. For example, disrupting the Acyl-CoA Synthetase Bubblegum Family Member 1(Acsbg1) gene in Treg cells has shown that their functional maintenance relies on intact mitochondrial fatty acid metabolism (119). This distinct metabolic preference makes the Th17/Treg axis an attractive therapeutic target; modulating the FAS/FAO balance may reshape immune responses and suppress autoimmune inflammation (113).

##### 3.1.1.2 Metabolic profiles of Th1 and Th2 cells

The differentiation and function of Th1 and Th2 cells are regulated by distinct lipid metabolic pathways. In Th1 cells, the cholesterol biosynthesis pathway and its precursor mevalonate promote differentiation and enhance IFN-γ production (120). Consistent with this, inhibition of this pathway using statins selectively suppresses the production of IFN-γ and IL-10 without affecting cell viability, underscoring the specificity of metabolic regulation in Th1 function (121). Moreover, fatty acid metabolism profoundly influences Th1 functional states. For instance, loss of the monounsaturated fatty acid *Scd2* activates type I interferon signaling in Th1 cells. This finding offers a novel metabolic–immune perspective for understanding the role of Th1 cells in antiviral defense and autoimmune inflammation (122).

In contrast, Th2 cell differentiation and function exhibit a stronger reliance on fatty acid metabolism. PPARγ is highly expressed during Th2 differentiation, and both PPARγ antagonism and inhibition of FAS significantly impair Th2 proliferation, differentiation, and secretion of signature cytokines such as IL-5 and IL-13 (123). Additionally, Th2 cells display high tolerance to lactate-rich environments, suggesting a metabolic preference for fatty acid oxidation(FAO) over glycolysis as an energy source (124, 125).

#### 3.1.2 CD8+T cells

Lipid metabolism serves as a central regulator of CD8^+^ T cell fate and function, profoundly influencing their efficacy in antitumor immunity (126). To meet the demands of proliferation and effector functions, activated CD8^+^ T cells not only uptake exogenous lipids but also engage in *de novo* lipogenesis (DNL) to sustain optimal effector responses (127, 128).

However, in the tumor microenvironment (TME), cholesterol can upregulate Cluster of Differentiation 36(CD36) on CD8^+^ T cells, leading to intracellular lipid accumulation, lipid peroxidation, and ferroptosis. These changes ultimately impair cytotoxicity and promote tumor progression (129). Notably, targeting CD36 can restore cytotoxic T lymphocyte (CTL) function and synergize with anti–PD-1 therapy (129).

On the other hand, memory CD8^+^ T cell (Tmem) persistence relies on highly efficient catabolic metabolism, particularly FAO, to maintain their quiescent state and metabolic flexibility. Similar to Treg cells, this process is independent of CPT1α (118, 130). Interestingly, Tmem cells depend less on extracellular fatty acid uptake. Instead, they utilize glucose taken up from the extracellular environment to support FAO and oxidative phosphorylation (OXPHOS) (131).

#### 3.1.3 γδTcells

Beyond the previously emphasized Th17 cells, IL-17-producing γδ T (γδ T17) cells are increasingly recognized for their pivotal role in autoimmunity. As a major subset of innate-like lymphocytes, γδ T17 cells can rapidly and abundantly produce IL-17 within hours upon stimulation by IL-1β and IL-23, independent of conventional T cell receptor signaling (132). In various models, including experimental autoimmune encephalomyelitis (EAE), psoriasis, and arthritis, γδ T17 cells have been identified as a critical early source of IL-17, often preceding the activation of Th17 cells (133).

Recent studies reveal that the differentiation and function of γδ T17 cells are highly lipid-dependent. In inflammatory settings such as psoriasis, these cells shift toward aerobic glycolysis and lipogenesis. They also uptake free fatty acids, such as palmitate, via the CD36 receptor, which further stimulates IL-17A production (134). Importantly, inhibiting acetyl-CoA carboxylase ACC1 disrupts *de novo* fatty acid synthesis in γδ T17 cells, reducing their lipid storage and IL-17A secretion capacity. This metabolic intervention has been shown to markedly ameliorate inflammation in a psoriasis model (135).

### 3.2 B cells and plasma cells

B cell activation and proliferation also require substantial lipids for membrane expansion. Specifically, sterol (cholesterol) and fatty acid synthesis, regulated by the SREBP pathway, are crucial for B cell activation, germinal center (GC) formation, memory B cell generation, and ultimate differentiation into antibody-secreting plasma cells (PCs) (22, 68). Genetic deletion of SCAP, a key protein in the SREBP signaling pathway, in B cells abolishes effective proliferation and reduces lipid raft structures, indicating the necessity of SREBP-mediated lipogenesis for B cell responses (68). During the GC reaction, B cells undergo intense proliferation and affinity maturation, a process also dependent on mitochondrial metabolism and FAO for energy (23). Differentiated PCs significantly upregulate DNL and cholesterol synthesis to provide phospholipids and cholesterol needed for constructing the extensive ER membrane system required for high-rate antibody secretion (24). Inhibiting fatty acid synthesis severely impedes PC differentiation and antibody secretion capacity (166). Thus, lipid metabolic reprogramming is fundamental to the high antibody production function of PCs.

### 3.3 Dendritic cells

When DCs are activated via pattern recognition receptors such as Toll-like receptors (TLRs), they undergo metabolic reprogramming. This process upregulates glycolysis and *de novo* lipogenesis (DNL), leading to lipid droplet accumulation. These changes enhance their immunogenicity, enabling efficient initiation of immune responses (136).

Specifically, the maturation of cDC1s, their expression of co-stimulatory molecules, and production of pro-inflammatory cytokines like IL-12 are crucial for initiating Th1-type immune responses (136). Furthermore, membrane cholesterol enrichment in cDC1s is key for forming effective antigen presentation platforms. Impaired cholesterol efflux specifically enhances the immunogenicity of cDC1s (137). Conversely, LXR agonists inhibit the maturation of cDC2s by promoting cholesterol efflux (80). However, excessive lipid accumulation, such as that induced by a high-fat diet, impairs the antigen presentation capacity and lymph node migration of CD11b+ cDC2s. This shifts their function towards inducing immune tolerance rather than immune activation (138, 139).

### 3.4 Macrophages

Macrophages can polarize into pro-inflammatory M1 or anti-inflammatory M2 phenotypes, a balance often disrupted in ADs (140). The classical model posits that M1 macrophages are characterized by high aerobic glycolysis and a disrupted TCA cycle, coupled with FAS activation, while M2 macrophages rely on an intact TCA cycle and OXPHOS fueled by FAO. Activation of nuclear receptors like PPARγ and LXR is key for driving M2 polarization (141, 142). However, recent studies challenge this clear dichotomy, suggesting human M2 macrophages may also rely on glucose metabolism, and highlighting potential off-target effects of commonly used FAO inhibitors like etomoxir, indicating a more complex metabolic landscape for macrophages than previously thought (143–145).

It is evident that the lipid metabolic preferences of adaptive immune cells form the metabolic basis for their functional specialization. From FAS-dependent pathogenic Th17 cells to FAO-preferring Treg cells, and from plasma cells requiring extensive lipid synthesis to support antibody production to dendritic cells whose antigen-presenting capacity is influenced by lipid accumulation, metabolic characteristics directly determine the nature and intensity of immune responses. However, the metabolic programs of immune cells do not exist in isolation; they are profoundly shaped by their tissue microenvironment. Variations in lipid abundance and composition across different tissues shape distinct T cell communities. For instance, the intestinal environment, rich in dietary lipids and microbially derived bile acids, promotes the differentiation and functional maintenance of Treg cells to sustain immune tolerance (146). The skin, abundant in ceramides (CER), cholesterol (CHOL), and free fatty acids (FFA), serves as a key reservoir for resident memory T cells (147). Nevertheless, when this lipid environment undergoes quantitative or qualitative alterations, it can transform into a pathological foundation that drives chronic inflammation and autoimmunity (148, 149). In the following section, we will explore how lipid metabolism is altered in the pathological context of autoimmune diseases.

## 4 Landscape of lipid metabolic dysregulation in autoimmune diseases

**Figure 3:**
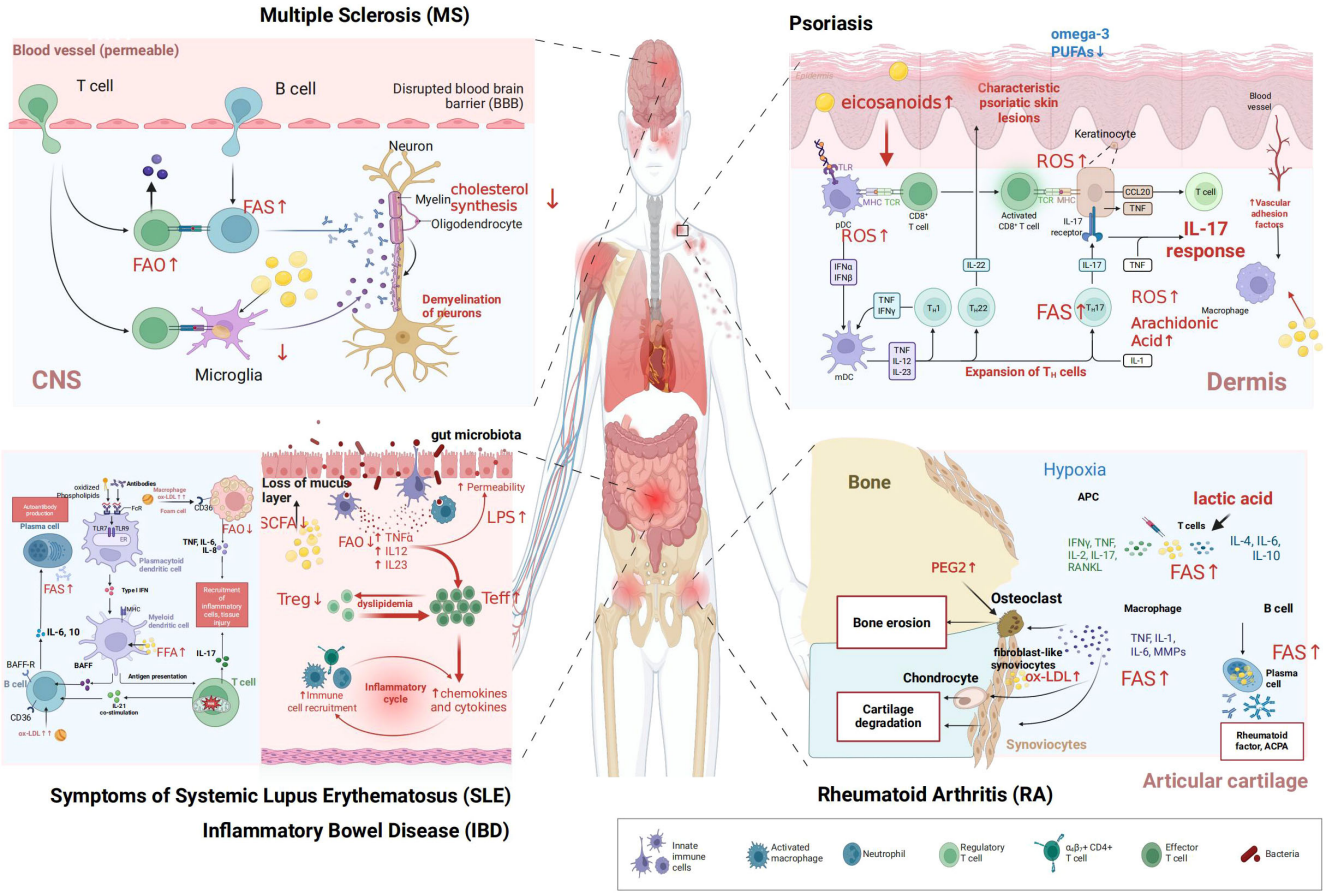
Dysregulated lipid metabolism in autoimmune diseases. This figure illustrates dysregulated lipid metabolism in key immune cells across five representative autoimmune diseases (1) MS: microglia, T cells, and B cells exhibit altered fatty acid oxidation (FAO), while oligodendrocytes show impaired cholesterol synthesis, contributing to neuronal demyelination (2) psoriasis: keratinocytes, T helper 17 (Th17) cells, plasmacytoid dendritic cells (pDCs), and macrophages display dysregulated arachidonic acid metabolism and reactive oxygen species (ROS) production, with reduced omega-3 polyunsaturated fatty acids (PUFAs), driving psoriatic skin lesion formation (3) SLE: autoreactive B cells, plasma cells, dendritic cells, and macrophages undergo abnormal FAO and interact with oxidized lipoproteins, facilitating autoantibody generation (4) IBD: effector T cells (Teff), regulatory T cells (Treg), and macrophages show disrupted FAO; coupled with gut microbiota dysbiosis (impacting short-chain fatty acids, SCFAs) and increased intestinal permeability, this fuels the inflammatory cycle (5) RA: fibroblast-like synoviocytes (FLS), osteoclasts, macrophages, T cells, and B cells exhibit dysregulated lipid metabolism (e.g., lactic acid accumulation, oxidized low-density lipoprotein (ox-LDL) involvement), leading to bone erosion and cartilage degradation. The legend depicts innate immune cells, activated macrophages, neutrophils, regulatory T cells, T helper subsets, effector T cells, and bacteria. ”Created in BioRender. yu, Y (2025). https://BioRender.com/21li3ok.

Lipid metabolic dysregulation is a common pathological feature of various autoimmune diseases (16). However, distinct immune cell subsets and tissue microenvironments in different diseases exhibit unique patterns of lipid metabolic reprogramming (**Figure 3**). As previously discussed, this reprogramming is not merely a passive consequence but actively contributes to disease pathogenesis. In this section, we will systematically outline the specific landscape of lipid metabolic dysregulation in five representative autoimmune diseases: SLE, RA, MS, IBD, and psoriasis. Furthermore, we will clarify how unique patterns of metabolic disturbance in different disease contexts contribute to their specific immunopathological characteristics (**Table 1**).

### 4.1 Systemic lupus erythematosus

SLE is a systemic autoimmune disease characterized by abundant autoantibody production and multi-organ involvement (150, 151). Its pathogenesis involves dysfunction of various immune cells, including T cells, B cells, and APCs, closely linked to profound lipid metabolic abnormalities (152).

CD4+ T cells, central drivers in SLE, exhibit significant lipid metabolic abnormalities (153, 154). Given the crucial role of mTORC1-driven lipogenesis in promoting Th17 cell differentiation (82), its dysregulation is particularly relevant to the pathogenesis of SLE. Concurrently, the immunoregulatory function of Treg cells is suppressed (151–153). Studies have confirmed that activation of mTORC1 precedes the onset of SLE and related comorbidities, indicating that this metabolic reprogramming acts as a key upstream event driving T cell functional imbalance (86). Concurrently, T cells in SLE commonly exhibit mitochondrial dysfunction. This includes hyperpolarized mitochondrial membrane potential, increased production of reactive oxygen species (ROS), abnormal morphology (such as enlargement), and reduced ATP generation (155). These alterations impair energy supply and affect epigenetic modifications like DNA methylation, leading to aberrant expression of autoreactive genes. Reducing lipid peroxidation/ROS and inhibiting T cell oxidative stress can alleviate lupus nephritis (LN) (156–158). Furthermore, lipid rafts contribute to SLE pathogenesis (159). Increased synthesis of cholesterol and glycosphingolipids (GSLs) in SLE T cells leads to excessive lipid raft aggregation on the plasma membrane, enhancing TCR signaling and lowering the activation threshold for autoreactive T cells (160, 161). Downregulating the transcription factor FLI1 reduces GSL synthesis, affecting SLE progression (79, 87, 162, 163). Recent studies confirm increased CD38 expression in SLE CD4+ T cells correlates with increased lipid rafts, and targeting CD38 to modulate ganglioside GM2 distribution alleviates SLE pathology (161).

Autoreactive B cells in SLE upregulate the scavenger receptor CD36 to increase exogenous lipid uptake (164), enhancing mitochondrial OXPHOS to fuel B cell activation, proliferation, and differentiation, thereby exacerbating disease (165). B cell differentiation into PCs depends on SREBP-mediated lipogenesis to support massive ER expansion. Inhibiting fatty acid synthesis ameliorates disease in lupus-prone mice (166, 167). Conversely, regulatory B cells (Bregs) in SLE are reduced in number and functionally impaired, exhibiting metabolic abnormalities like mitochondrial depolarization and elevated ROS (168).

Macrophages and DCs also play significant roles in SLE, particularly LN (169). In LN models, renal macrophages exhibit impaired phagocytic function, failing to clear apoptotic cells and immune complexes effectively. Excess lipid peroxides can activate macrophage inflammation (170), while their phagocytic capacity is impaired due to downregulated CPT1α and reduced fatty acid metabolism (171, 172). Additionally, high levels of type I interferon persistently induce upregulation of the scavenger receptor SR-A1 on macrophages, promoting uptake of oxidized low-density lipoprotein (oxLDL) and foam cell formation, accelerating atherosclerosis—a mechanism underlying the high cardiovascular risk in SLE patients (169). Research on DCs in SLE is less extensive. In LN, DCs infiltrate the kidney and amplify inflammation (173). Recent studies show that accumulated cholesterol metabolite farnesyl pyrophosphate (FPP) within DCs of SLE model mice promotes their activation via mitochondrial remodeling (174).

In summary, in SLE, mTOR-driven metabolic anomalies in T cells, excessive lipid uptake by B cells, and macrophage dysfunction drive premature immune cell activation, amplifying metabolic disturbances and providing the basis for autoantibody production and multi-organ inflammatory damage.

### 4.2 Rheumatoid arthritis

RA is an autoimmune disease characterized by chronic synovial inflammation and joint cartilage/bone erosion (175).The pathological core lies in the pathogenic interaction between immune and stromal cells within the synovial microenvironment, wherein lipid metabolism serves a dual role as both an energy source and a signaling mechanism.

The inflamed RA synovium constitutes a unique metabolic microenvironment characterized by hypoxia and high lactate concentrations (176). High lactate is taken up by synovial CD4+ T cells via the transporter Solute Carrier Family 5 Member 12(SLC5A12), which inhibits their glycolysis and paradoxically drives FAS (177, 178). This lactate-induced increase in FAS is a key metabolic basis for T cell differentiation towards the pathogenic Th17 phenotype and IL-17 production. This mechanism directly links accumulated this local metabolite (lactate) to T cell pathogenicity (179, 180). Concurrently, excess FAs lead to LD deposition and upregulate T cell migration programs, facilitating immune cell infiltration and exacerbating synovitis and joint destruction (91, 176). Fibroblast-like synoviocytes (FLS) are major effectors of joint destruction in RA. RA-FLS exhibit upregulated FASN expression leading to fatty acid accumulation (181, 182). These excess FAs enhance phosphorylation of DRP1 protein, inducing mitochondrial fission, which increases ROS production and activates pro-inflammatory and pro-survival pathways like PI3K/mTOR/NF-κB, ultimately conferring the aggressive, cartilage-destructive phenotype to FLS (92). The RA synovium is infiltrated by numerous macrophages. Active RA is dominated by MerTK-negative (MerTK−) macrophages secreting pro-inflammatory cytokines, while remission phases enrich MerTK-positive (MerTK+) macrophages with repair functions (93). The latter can effectively produce SPMs (e.g., resolvins), inducing FLS repair responses and maintaining joint homeostasis (93). A hallmark pathological change in RA is bone erosion caused by excessive osteoclast activation. Osteoclast differentiation is regulated by cytokines like Receptor Activator of Nuclear Factor Kappa-B Ligand(RANKL) (183). It is also closely linked to cholesterol metabolism (184). Statins, besides lowering lipids, can inhibit RANKL expression and inhibit osteoclast precursor differentiation, suggesting potential value for bone protection in RA (185). The hypoxic synovial microenvironment in RA initially induces lipid synthesis in T cells, and this metabolic reprogramming in turn drives their pathogenic phenotype and tissue infiltration capacity, illustrating the vicious cycle between metabolic abnormality and immune activation.

In the hypoxic joint microenvironment of RA, lactate accumulation drives lipid metabolic reprogramming in T cells and FLS. This reprogramming promotes the differentiation of pathogenic Th17 cells, enhances the invasiveness of FLS, and disrupts bone homeostasis, thereby synergistically exacerbating synovial inflammation and joint destruction. Hypoxia and lipid signaling act cooperatively within the RA joint microenvironment to jointly drive disease progression. Specifically, the hypoxic synovial milieu initially induces lipid synthesis in T cells, and this metabolic reprogramming in turn drives pathogenic T cell phenotypes and enhances tissue infiltration capacity. This process exemplifies a vicious cycle between metabolic dysregulation and immune activation.

### 4.3 Multiple sclerosis

MS is an autoimmune disease characterized by chronic central nervous system (CNS) inflammation, demyelination, and neurodegeneration (186). Patients with MS exhibit significant alterations in lipid metabolism, including changes in levels of low-density lipoprotein (LDL), high-density lipoprotein (HDL), apolipoproteins, and oxysterols. These metabolic abnormalities correlate with clinical disease activity, although their causal relationship remains incompletely defined (187). Given that myelin itself is a lipid-rich structure, lipid metabolic disturbances occupy a central position in MS pathogenesis; lipid molecules are both targets of myelin destruction and inflammatory mediators affecting immune function.

Myelin, formed by extensions of oligodendrocyte membranes wrapping around axons, is rich in lipids (constituting ~70-80% of dry weight) (188). Therefore, oligodendrocytes must maintain high lipogenic capacity for myelination during development and effective remyelination after damage (189). The failure of remyelination in MS is partly due to impaired differentiation of oligodendrocyte precursor cells into mature oligocytes. Consequently, these cells face a severe deficit in their lipid synthesis capacity, which is required for producing the vast amounts of lipids essential for myelin production (189). Altered myelin composition due to lipid dysmetabolism may affect its stability and increase its immunogenicity, potentially triggering immune attack (190). In MS demyelinating lesions, microglia and infiltrating macrophages phagocytose myelin debris, leading to lipid overload and foam cell formation (191). This lipid accumulation triggers sustained inflammation and inhibits transition to a pro-repair phenotype, hindering remyelination. The transcription factor Interferon Regulatory Factor 5(IRF5) is key for regulating myelin debris degradation and cholesterol homeostasis; IRF5 deficiency leads to inadequate degradation in lysosomes, causing abnormal accumulation of LDs and cholesterol crystals, exacerbating disease (191). Activity of the fatty acid elongase ELOVL6 has also been found to promote inflammatory foam cell formation (192). Changes in mitochondrial lipid metabolism have been identified in CD4+ T cells from MS patients (193). In the experimental autoimmune encephalomyelitis (EAE) mouse model, Treg cells infiltrating the CNS heavily rely on CPT1α-mediated FAO to maintain their function; enhancing Treg FAO capacity is considered a potential therapeutic strategy (194). Furthermore, the lipid mediator maresin-1 (MaR1) can reduce Th1 cells, increase Tregs, and suppress pro-inflammatory cytokines (98, 195), also reducing immune cell infiltration, accelerating inflammation resolution, and delaying disease progression in EAE models (102). CD4+ T cells from relapsing-remitting MS (RRMS) patients exhibit dysregulated LXR-mediated lipid metabolism. While LXRβ expression is upregulated, downstream target gene expression is downregulated, leading to increased membrane cholesterol and decreased GSLs. This altered lipid raft composition is thought to enhance T cell reactivity, promote IL-17 production, and exacerbate neuroinflammation (99). Recently, a lipid kinase was found to promote Th17 differentiation via the mTORC1/STAT3 pathway, contributing to EAE progression (103). A clinical study profiling lipid metabolic reprogramming in immune cells of MS patients is ongoing (NCT04053374). Metabolic reprogramming endows pathogenic T cells with strong migratory and pathogenic capacity, enabling CNS invasion and inflammatory cytokine release; these cytokines, in turn, exacerbate lipid metabolic reprogramming in CNS immune cells and damaged cells, leading to myelin and neuronal injury.

### 4.4 Inflammatory bowel disease

IBD, including Crohn’s disease (CD) and ulcerative colitis (UC), involves a loss of tolerance to intestinal microbiota in genetically susceptible individuals, resulting in chronic gut inflammation. A nationwide study suggests abnormal lipid profiles in CD and UC patients (196). Lipid metabolism plays multifaceted roles in IBD, regulating intestinal barrier function, microbial homeostasis, and immune responses (197).

A healthy gut microbiota ferments dietary fiber to produce short-chain fatty acids (SCFAs), notably butyrate (198). Butyrate is a primary energy source for colon epithelial cells and a potent histone deacetylase (HDAC) inhibitor (199).Through this epigenetic mechanism, it promotes Treg cell differentiation while suppressing pro-inflammatory responses, ultimately maintaining intestinal immune tolerance (146). Patients with IBD commonly exhibit gut microbiota dysbiosis, characterized particularly by a reduction in butyrate-producing bacteria. This leads to decreased levels of butyrate, which is recognized as a key driver of inflammation in IBD (200). In ulcerative colitis, lipidomic analyses have revealed that alterations in triglyceride and phospholipid levels are closely linked to the pathogenesis, progression, and treatment response of the disease (201). CD patients exhibit a unique “creeping fat” phenomenon: mesenteric adipose tissue abnormally proliferates and wraps around the intestine (202). Creeping fat is infiltrated by immune cells and secretes high levels of adipokines like pro-inflammatory leptin. Interestingly, macrophages in CD creeping fat often exhibit M2 polarization promoting tissue remodeling, but the hyperplastic fat itself becomes a reservoir for inflammatory cells (203). Intestinal lamina propria macrophages are sentinels for mucosal immune homeostasis. In inflamed IBD gut, their metabolic reprogramming is characterized by significant downregulation of the anti-inflammatory nuclear receptor PPARγ and FAO pathways (204). This impairs their ability to polarize towards an M2 phenotype, preventing effective inflammation suppression and repair, thereby exacerbating gut damage. The probiotic Fecalibacterium prausnitzii can reprogram macrophage energy metabolism, guiding them towards M2 polarization and alleviating intestinal fibrosis in CD patients (205). Another study found Fatty Acid-Binding Protein 5(FABP5) upregulated in IBD macrophages, potentially exerting anti-inflammatory effects by preventing M1 polarization (206). IBD patients have impaired intestinal barrier function, associated with sphingolipid metabolism abnormalities, such as potentially elevated pro-inflammatory S1P and imbalances in ceramides necessary for barrier integrity (207). Furthermore, bile acids, synthesized by the liver and modified by microbiota, regulate intestinal immunity by activating receptors like Farnesoid X Receptor (FXR) and Takeda G protein-coupled Receptor 5 (TGR5); their dysregulation also contributes to IBD pathogenesis (208, 209). In summary, immune cells in IBD exhibit significant metabolic reprogramming, including abnormalities in glucose/lipid metabolism and imbalanced microbiota-immune cell interactions, collectively driving and sustaining pathological gut inflammation.

### 4.5 Psoriasis

Psoriasis is a chronic inflammatory skin disease characterized by hyperproliferation of keratinocytes and immune cell infiltration (210). Its core pathological mechanism involves overactivation of the IL-23/Th17 axis (210). IL-17 production in psoriasis is not restricted to Th17 cells. Within psoriatic lesions, IL-17-producing skin γδ T cells are also pivotal in driving psoriasiform dermatitis. Moreover, the IL-36 signaling pathway plays a central and unique role in amplifying the IL-23/IL-17/IL-22 inflammatory axis and promoting disease progression (211). The pathogenesis of psoriatic lesions involves not only dysregulated immune-keratinocyte crosstalk but also cellular metabolic reprogramming. Critically, Th1/Th17 and Th2 cytokines exert divergent influences on lipid metabolism in differentiating keratinocytes. This metabolic influence is proposed as a key mechanism underlying the dysfunction of the skin barrier in psoriasis (212).Concurrently, cytokines from DCs prompt keratinocyte hyperproliferation and production of more chemokines/cytokines, further attracting immune cell infiltration, forming a vicious cycle (213, 214). Lipid metabolic abnormalities in psoriasis manifest at both local (skin) and systemic levels, accelerating disease progression by altering immune cell phenotypes and functions (215).

Healthy skin barrier function relies on a precise ratio of lipids (ceramides, cholesterol, free fatty acids) in the stratum corneum (108). Psoriatic lesions exhibit significant barrier defects and abnormal lipid profiles (216, 217), characterized by reduced levels of anti-inflammatory omega-3 polyunsaturated fatty acids (PUFAs) and elevated levels of pro-inflammatory arachidonic acid (AA). AA is the precursor for potent pro-inflammatory eicosanoids (e.g., prostaglandins, leukotrienes), which directly drive skin inflammation (218). Supplementing omega-3 PUFAs to modulate skin inflammation may have clinical significance (219, 220).

Moreover, psoriasis is a systemic inflammatory disease. A recent study suggests a significant association between elevated triglyceride levels and the risk of psoriasis (221). Furthermore, patients with psoriasis have a markedly increased risk of cardiovascular diseases, including atherosclerosis (109). A key link underlying this comorbidity is lipid metabolic dysregulation. Systemic inflammation causes dyslipidemia (222) and promotes monocyte/macrophage infiltration into vessel walls. In the inflammatory microenvironment, these macrophages avidly take up oxidized lipids, transforming into foam cells that initiate and accelerate atherosclerosis, a mechanism similar to that in SLE (223). Similarly, immune cells in psoriasis patients exhibit significant oxidative stress, leading to lipid peroxidation and the production of pro-inflammatory mediators (224, 225) (see Section 3.1). Moreover, these ROS-dependent lipid mediators activate pro-inflammatory signaling, promote Th1/Th17 differentiation, and stimulate keratinocytes (110). Simultaneously, Th17/Treg cells display a functional imbalance analogous to that observed in SLE, which disrupts a critical immunoregulatory equilibrium (88).Meanwhile, pathogenic Th17 cells favor aerobic glycolysis and lipogenesis, while Treg FAO is suppressed in the inflammatory environment, leading to insufficient immunosuppressive function (88).In psoriasis patients, levels of Th2-related cytokines or cell populations are reduced and negatively correlate with disease severity (226). Diagnostically, the combination of Th2+Treg cell ratio and adiponectin levels enables high-precision prediction of psoriasis (227). Mechanistically, recent research identifies Th2 immunity as a key tissue checkpoint that suppresses skin autoimmunity and maintains lipid homeostasis, conferring resistance to psoriasis by sustaining LXR/PPARγ-mediated fatty acid metabolism via STAT6 signaling (228).

A unifying theme across these diseases is the central role of “metaflammation” within target organs. In summary, although shared features of lipid metabolic dysfunction—such as hyperactive mTOR signaling and mitochondrial dysfunction—exist across autoimmune diseases, each disorder exhibits unique metabolic characteristics shaped by its specific tissue microenvironment. This spectrum ranges from lipid raft aggregation in T cells in SLE, to lactate-driven fatty acid synthesis in the RA synovium, and from imbalanced myelin lipid metabolism in MS to disrupted host-microbiota interactions in IBD. Critically, in affected tissues—be it the synovium in RA, the CNS in MS, the gut in IBD, or the skin and vasculature in psoriasis—the local metabolic milieu (e.g., lactate, lipids, and microbial metabolites) is not a passive backdrop but an active participant. It shapes the metabolic programs and functional phenotypes of immune cells, which in turn secrete inflammatory factors that further worsen the local metabolic environment, establishing a self-sustaining and amplifying pathological circuit (**Table 2**). This understanding underscores that effective therapeutic strategies may need to target not only the immune cells themselves but also strive to normalize the metabolic microenvironment of diseased tissues, thereby breaking this vicious cycle.

**Table 2:** Summary of dysregulated lipid metabolism in major autoimmune diseases. ① Preclinical: Studies conducted before human clinical trials (e.g., pharmacology, toxicology); ② Clinical: Trials in human subjects for safety and efficacy evaluation.

Disease	Key immune cell(s)	Core lipid metabolic abnormalities	Pathological consequences	Preclinical & clinical evidence	References
SLE	T cells, B cells, Macrophages, Dendritic Cells	**T cells**: Enhanced mTOR-driven glycolysis & FAS; **B cells**: Enhanced CD36-mediated lipid uptake; **Macrophages**: Impaired FAO; **DCs:** Altered mitochondrial metabolism	Broken immune tolerance; Autoantibody production; Accelerated atherosclerosis	**Preclinical:** Rapamycin improves mouse models; CD36 knockout alleviates disease. **Clinical:** Fluvastatin reduces disease activity; Sirolimus effective.	(86–90)
RA	T cells, Fibroblast-like synoviocytes (FLS), Macrophages	**T cells/FLS**: Enhanced fatty acid synthesis; **Macrophages**: Deficient SPM synthesis	Chronic synovitis; Cartilage destruction & bone erosion	**Preclinical:**: FASN inhibition alleviates joint damage. **Clinical:**: Inhibiting T cell FAS reverses pathogenicity; Statins improve outcomes.	(91–97)
MS	Microglia/Macrophages, T cells	**Myelin lipids**: Overload & impaired degradation; **Cholesterol efflux**: Impaired; **T cells**: Dysregulated LXR signaling, enhanced FAS	CNS demyelination; Failed remyelination; Neurodegeneration	**Preclinical:**: LPA1 antagonist, PIKFYVE inhibition, MaR1 effective in EAE. **Clinical:**: LXR agonist inhibits Th17; Oleic acid restores Treg function.	(98–102)
IBD	Macrophages, T cells, Epithelial cells	**Gut microbiota**: Reduced SCFA production; **Macrophages**: Impaired FAO; **Epithelial barrier**: Ceramide deficiency	Intestinal barrier dysfunction; Chronic inflammation; Treg impairment	**Preclinical:**: FABP5 inhibitor, PPARγ agonists ameliorate colitis. **Clinical:**: T cell lipid raft abnormalities in CD; Enhancing FAO reverses pathogenic Trm cells.	(103–107)
Psoriasis	Keratinocytes, T cells, Macrophages	**Keratinocytes**: Altered epidermal lipid synthesis; Systemic: Dyslipidemia; **Immune cells**: Increased pro-inflammatory eicosanoids	Skin barrier defect; Keratinocyte hyperproliferation; Increased cardiovascular risk	**Preclinical:**: Suppressed Treg FAO; ACC1 deficiency ameliorates model. **Clinical:**: Cholesterol efflux capacity (CEC) inversely correlates with activity; LXR/PPARγ activation beneficial.	(108–112)

Bold terms highlight key categorical elements within the table, including specific immune cell types, pivotal lipid pathways or components, critical anatomical structures, and the categories of scientific evidence.

Immune cells (e.g., T cells, DCs) and disease-specific cell types (e.g., FLS, Keratinocytes).Core lipid components and pathways (e.g., Myelin lipids, Cholesterol efflux).Critical anatomical structures (e.g., Epithelial barrier).Categories of research evidence (Preclinical, Clinical).

## 5 Therapeutic strategies targeting lipid metabolism: from drugs to clinic

Given the central driving role of lipid metabolism in ADs, targeting related pathways has become an highly attractive new therapeutic direction. Strategies can be broadly categorized into three groups: repurposing approved drugs for immunomodulatory effects, developing new drugs against emerging metabolic targets, and modulation via dietary and microbial interventions.

### 5.1 Novel immunomodulatory uses of marketed drugs

Some metabolic modulators already widely used in the clinic possess unexpected immunomodulatory effects, offering a promising path for “drug repurposing”.

#### 5.1.1 Statins

Statins are HMGCR inhibitors primarily used for cholesterol lowering. However, their potential in treating ADs stems largely from pleiotropic anti-inflammatory effects (238). By inhibiting the mevalonate pathway, statins not only reduce cholesterol synthesis but, importantly, reduce the production of isoprenoid intermediates (e.g., FPP, GGPP). These intermediates are required for the prenylation of small GTPases (e.g., Rho, Ras), which are key nodes in inflammatory signaling pathways (239). Thus, statins can inhibit T cell activation, skew them from pro-inflammatory Th1/Th17 towards anti-inflammatory Th2 phenotypes (212), and inhibit DC maturation and antigen presentation capacity (240). In the realm of clinical trials, statins such as simvastatin (MS-STAT2 trial, NCT03896217) for MS and atorvastatin (NCT00356473, NCT04177173) for RA have demonstrated the potential to reduce disease activity and slow progression (241, 242). However, the results are inconsistent across studies, with some failing to observe significant beneficial effects (243). Efficacy in SLE is also debated. A retrospective study found adding atorvastatin to standard therapy improved immune function and disease activity indices in mild-to-moderate active SLE patients (244), while another found atorvastatin had no significant effect in MRL/lpr mice. However, *in vitro* experiments confirmed statins inhibit splenic B cell proliferation, suggesting potential for SLE (245). This indicates statin efficacy may be disease- and patient-specific.

#### 5.1.2 S1P receptor modulators

Fingolimod, siponimod, and ozanimod are functional antagonists of S1P receptors (246). S1P binding to its receptor S1PR1 is necessary for lymphocyte egress from lymph nodes into circulation. These drugs, by binding S1PR1 and inducing its internalization and degradation, sequester lymphocytes in lymph nodes, preventing autoreactive lymphocytes from migrating to target organs like the CNS (43). Fingolimod, the first oral drug approved for MS, significantly reduces relapse rates and delays disability progression (247). Later-developed siponimod and ozanimod have higher receptor selectivity (248). Given their broad immunomodulatory effects, the potential of S1P receptor modulators in other ADs is being actively explored. For example, ozanimod is under investigation for UC (NCT05369832). Trial criteria sometimes mention RA and SLE, hinting at potential applications (249).

#### 5.1.3 Fibrates

Fibrates like fenofibrate are PPARα agonists used primarily for hypertriglyceridemia. By activating PPARα, these agents drive metabolic reprogramming in T cells. This metabolic shift enhances FAO, thereby alleviating inflammation associated with ADs (250). Although research in ADs is early-stage, a trial plans to explore fenofibrate’s role in preventing chemotherapy-induced neuropathy (NCT07025005), indirectly suggesting potential utility in neuroinflammatory diseases like MS.

#### 5.1.4 PPARγ agonists

Thiazolidinediones (TZDs) like pioglitazone are potent PPARγ agonists. They promote macrophage polarization to the M2 anti-inflammatory phenotype, inhibit inflammatory pathways, and may improve skin barrier function, offering theoretical benefits in RA, IBD, and psoriasis. However, first-generation TZDs, affecting systemic lipid/glucose metabolism, have side effects (weight gain, edema) limiting use in non-diabetic populations (251). Research focuses on developing newer, more selective PPARγ agonists with better safety. A drug interaction study assessed pioglitazone pharmacokinetics in IBD patients (NCT02371603). Oral pioglitazone significantly improved clinical measures in secondary progressive MS patients without serious adverse events (NCT00242177) (252). Pioglitazone treatment in young female SLE patients significantly decreased inflammatory markers (NCT01322308) (253). In RA patients, pioglitazone significantly reduced disease activity and CRP levels, improved lipid profiles, and was well-tolerated (NCT00554853) (254), supported by other studies (NCT00763139; NCT02338899) (255, 256).

### 5.2 Emerging targets and drugs in clinical development

A range of new drugs targeting more specific nodes in immunometabolic pathways are under development, showing great therapeutic promise.

#### 5.2.1 mTOR inhibitors

Sirolimus (rapamycin) and its analog everolimus are specific inhibitors of mTORC1. As mentioned, mTORC1 is hyperactivated in pathogenic T cells in SLE and RA. By inhibiting mTORC1, these drugs can reshape T cell balance: inhibiting Th1 and Th17 cell differentiation while promoting Treg cell expansion and function (257, 258). Several clinical trials are currently evaluating mTOR inhibitors. A Phase II trial (SIRIUS, NCT04582136) for active SLE is ongoing. Another early-phase trial at the US NIH explores sirolimus use in pediatric patients with autoimmune cytopenias related to SLE and RA, among others (NCT00392951). A Phase II trial for IgG4-related disease (IgG4-RD, NCT05746689) is not yet recruiting.

#### 5.2.2 PCSK9 Inhibitors

PCSK9 is a key protein regulating cholesterol metabolism by mediating LDLR degradation. Initially developed for lipid lowering, PCSK9 inhibitors are being explored for ADs due to anti-inflammatory effects. A Mendelian randomization study suggested PCSK9 inhibition significantly lowers SLE risk but may increase asthma and CD risk, with effects differing from HMGCR inhibitors (245). A phase II trial is assessing PCSK9 inhibitor effects on islet function and inflammation markers (e.g., hs-CRP, IL-6) in type 1 diabetes (NCT05641753), results pending.

### 5.3 Frontier directions in preclinical research

At the basic research level, numerous novel lipid metabolic targets are being discovered and validated (**Table 3**).

**Table 3:** Targeting lipid metabolism: a summary of therapeutic strategies for autoimmune diseases.

Therapeutic class	Representative agents	Primary metabolic target(s)	Key immunomodulatory effects	Targeted disease(s)	Development stage & key clinical trial ID(s)
HMG-CoA Reductase Inhibitors	Simvastatin, Atorvastatin	HMGCR/Mevalonate Pathway	Inhibits T cell protein prenylation; reduces Th1/Th17 differentiation	MS, RA, SLE	Phase II (MS - NCT03896217); Phase II (Graves’ Disease - NCT03110848); Phase II (RA - NCT00555230); Phase IV (RA - NCT04177173); Phase IV (RA - NCT00356473); Phase II (SLE - NCT00519363); Phase II (SLE - NCT00432354); Observational (RA/SLE - NCT01180361, recruiting).
S1P Receptor Modulators	Fingolimod, Ozanimod	S1PReceptor1 (S1PR1)	Sequesters lymphocytes in lymph nodes, preventing migration to inflammatory sites	MS, UC	Approved for MS (Fingolimod, Ozanimod, Siponimod) and UC (Ozanimod, Etrasimod); Hundreds of completed/ongoing trials (Phases III-IV, post-marketing surveillance).
mTOR Inhibitors	Sirolimus (Rapamycin)	mTORC1	Inhibits Th1/Th17 differentiation; promotes Treg expansion and function	SLE,ALPS, IgG4-RD	Phase II (SLE, NCT04582136, recruiting); Phase II (ALPS - NCT00392951); Phase II (IgG4-RD, NCT05746689, not yet recruiting).
Nuclear Receptor Agonists (PPAR)	Pioglitazone (PPARγ), Fenofibrate (PPARα)	PPARγ, PPARα	Promotes M2 macrophage polarization; enhances FAO; exerts anti-inflammatory effects	RA, IBD, MS	Phase II (SLE - NCT02338899); Phase IV (SLE - NCT01322308); Phase III (RA - NCT00554853); Phase II (RA - NCT00763139); Phase I (MS - NCT00242177); Phase IIa (IBD - NCT0594058)
Nuclear Receptor Agonists (LXR)	T0901317 (Preclinical), RGX-104	LXRα, LXRβ	Promotes macrophage cholesterol efflux; inhibits pro-inflammatory gene expression	SLE, MS	Preclinical (259–262)
FAS/ACC Inhibitors	TVB-2640 (FASN inhibitor), TOFA (ACC inhibitor)	FASN, ACC1	Inhibits pathogenic Th17 differentiation and autoantibody production by autoreactive B cells/plasma cells	RA,SLE, Psoriasis	Preclinical (8, 115, 181, 263–265)
Pro-Resolving Mediators	Resolvins, Protectins (SPMs)	GPCRs(e.g., GPR32)	Actively promote inflammation resolution; enhance efferocytosis	SLE,RA, IBD	Preclinical (98, 266–283)
Lipid Uptake Modulators	CD36 inhibitors	CD36Scavenger Receptor	Blocks lipid uptake in macrophages and B cells, preventing foam cell formation/activation	SLE, RA, MS	Observational Study (NCT01180361) (284); Preclinical (164, 285–289)

#### 5.3.1 Fatty acid synthesis inhibitors (FASN/ACC inhibitors)

Directly targeting the upregulated FAS pathway (e.g., FASN or ACC1) in pathogenic Th17 cells is a direct strategy to inhibit these cells (263). Various FASN and ACC inhibitors are in development, primarily for cancer and metabolic dysfunction-associated steatohepatitis (MASH). Inhibiting FASN-mediated lipid metabolism in B cells alleviates lupus in mice (166). The FASN inhibitor TVB-2640 can activate macrophages and DCs and significantly ameliorate imiquimod (IMQ)-induced lupus in mice (264), showing great potential, particularly in SLE.

#### 5.3.2 Lipid uptake and transport modulators (CD36, FATP modulators)

Targeting the scavenger receptor CD36 may prevent lipid overload in macrophages and B cells, blocking key steps in SLE-related atherosclerosis and autoreactive B cell activation (164, 285). PPARγ activation can upregulate CD36-mediated FAO, enhancing Treg responses, potentially beneficial for treating inflammation and ADs (290). In the RA synovium, CD36 promotes pro-inflammatory macrophage polarization and IL-1β/TNF-α release via lipid uptake. An observational study is analyzing whether plasma from SLE/RA patients affects CD36 expression on monocytes (NCT01180361), providing basis for intervention studies. In EAE, CD36 is required for myelin debris uptake by macrophages/microglia; its pharmacological inhibition worsened neuroinflammation and disease severity (286).

#### 5.3.3 Specialized pro-resolving mediators

SPMs, including resolvins, protectins, and maresins, are endogenous lipid mediators derived from omega-3 PUFAs. They do not passively suppress inflammation but actively initiate and coordinate the resolution of inflammation (10). SPMs restore tissue homeostasis by inhibiting neutrophil infiltration, enhancing macrophage clearance of apoptotic cells (efferocytosis), and promoting tissue repair (291). This “pro-resolution pharmacology” represents a novel therapeutic concept, shifting from anti-inflammatory to pro-resolving. Furthermore, SPM levels correlate with RA disease activity, and they directly inhibit pathogenic T cell proliferation by precisely regulating the Th17/Treg balance (254). This regulatory effect may involve the antagonism of key pro-inflammatory signals. For instance, in RA, γδ T cells represent a significant source of IL-17, and their function can conversely be enhanced by mediators such as prostaglandin E2 (PGE2) (267). This marks a transition in lipid metabolism research from correlative observation to functional target development. Exogenous administration of specialized pro-resolving mediators (SPMs) has demonstrated potent therapeutic efficacy in preclinical studies across various autoimmune disease models, including RA and MS, suggesting significant potential for clinical translation (98).

#### 5.3.4 LXR agonists

LXR agonists effectively promote macrophage cholesterol efflux and exert potent anti-inflammatory effects, making them ideal candidates for inflammatory diseases like atherosclerosis (292). Targeting T cell LXRβ improved disease severity in an MS model (259). An LXR inverse agonist, SR9243, alleviated RA by modulating macrophage metabolism (260). However, a major challenge is that LXR agonists induce SREBP-1c, leading to increased hepatic lipogenesis and steatosis (293). This off-target effect hinders clinical translation. Research focuses on developing tissue-specific or pathway-selective LXR agonists to avoid liver side effects while retaining anti-inflammatory benefits (294). An LXR agonist, RGX-104, has undergone Phase I trials in advanced cancer (NCT02922764), proving druggability and providing experience for future AD applications.

### 5.4 Therapeutic potential of dietary and microbial interventions

#### 5.4.1 Omega-3 PUFA supplementation

Dietary intervention is a direct means to modulate lipid metabolism. Diets rich in omega-3 PUFAs (e.g., EPA, DHA from fish oil) alter the body’s fatty acid profile, shifting eicosanoid synthesis from the pro-inflammatory omega-6 pathway towards producing anti-inflammatory or pro-resolving SPMs (295). Omega-3 supplementation modulates B cell differentiation in lupus-prone mice (296), reduces autoantibody production and immune complex deposition, and blocks interferon and chemokine gene expression in lupus (297, 298). Numerous clinical studies (including the large VITAL trial) have evaluated omega-3 supplements for preventing/treating RA, SLE, psoriasis, CD, etc., generally showing benefits in reducing disease activity (220, 299).

#### 5.4.2 Gut microbiota modulation

Targeting the gut microbiota is another frontier for modulating host metabolism and immunity. Strategies like probiotics, prebiotics, or fecal microbiota transplantation (FMT) can reshape gut microbial structure, increasing abundance of beneficial bacteria like butyrate producers, showing great promise in IBD treatment (300). Butyrate enhances gut barrier function and promotes Treg differentiation, significantly improving joint inflammation in the collagen-induced arthritis (CIA) model (301–303). Gut microbiota metabolites are key mediators linking nutrition and immunity: SCFAs promote Treg differentiation and enhance the gut immune barrier (304), while bacterially modified bile acids influence immune cell differentiation via receptor-mediated mechanisms (305–307). These findings promote integrated intervention strategies, e.g., using traditional Chinese medicine active components to modulate gut microbiota metabolism, potentially enabling multi-target immunometabolic regulation for chronic diseases.

### 5.5 Emerging therapeutic platforms and metabolic intersections

The convergence of emerging technologies with metabolic regulation is opening new avenues for modulating immunometabolic homeostasis to treat complex diseases. CAR-T therapy is being enhanced by metabolic reprogramming techniques to promote an FAO-dependent memory-like phenotype, preventing T cell exhaustion and enabling long-term survival/function in nutrient-poor, hypoxic environments, significantly improving efficacy. CAR-T cell therapy for ADs like SLE has entered clinical investigation (308). For example, CD19-CAR T cell therapy substantially inhibits key pathways in SLE, upregulating lipid metabolism-related pathways compared to rituximab and belimumab (309). In synthetic immunology, engineered probiotics designed to exploit APC metabolism, activating HIF-1α in DCs to produce lactate and inhibit autoreactive T cells, have been developed (310). In IBD models, pH-sensitive nanoparticle carriers can target butyrate delivery to inflamed gut areas, promoting Treg differentiation and barrier repair while reducing systemic side effects (311). These innovative cross-disciplinary technologies hold promise to revolutionize immunotherapy.

## 6 Conclusion

This review systematically elucidates the central role of lipid metabolism in immune regulation. The research paradigm has shifted from viewing lipids as passive structural and energy molecules to recognizing their active roles as signaling regulators and determinants of immune cell fate. Lipid reprogramming is a necessary condition for immune cell functional differentiation (80). It can also be induced by inflammatory factors (141). This forms a self-amplifying positive feedback loop, which acts as a core regulatory node driving disease initiation, progression, and chronicity. In ADs, distinct metabolic dysregulation—such as glycolytic hyperactivation in SLE, lactate-induced lipogenesis in RA, and myelin lipid overload in MS—characterizes each disorder. More importantly, these metabolic vulnerabilities present unprecedented opportunities for developing novel, more targeted therapeutic strategies. By reprogramming metabolism rather than using broad suppression, we can normalize immune function and restore homeostasis, representing a profound shift in the treatment philosophy for ADs.

A core paradox exists in targeting lipid metabolism for ADs: many effective drug targets (e.g., HMGCR, FASN) are essential for normal immune cell function (312). Why does inhibiting these pathways treat disease? Could it cause immunosuppression or metabolic toxicity? How to balance efficacy and safety? We posit that pathogenic immune cells in ADs exist in a state of metabolic hyperactivation, far exceeding normal homeostatic levels. The therapeutic goal is not complete pathway blockade but rather modulating runaway metabolic activity back to the normal homeostatic setpoint (313). The concept of “immunometabolic normalization” rather than “inhibition”—highlights the need for drugs with an appropriate therapeutic window. Such agents should effectively suppress pathological hyperactivation while avoiding excessive impairment of the normal metabolism essential for protective immunity.

Despite remarkable progress, challenges remain: while the association between lipid metabolic abnormalities and ADs is clear, precise causal chains need full elucidation. Determining the initiating factors and key nodes is crucial for identifying optimal intervention targets. Lipid metabolic regulation exhibits significant heterogeneity across diseases, patients, tissue microenvironments, and even cell subsets. For instance, inhibiting pathways like mTORC1 or FASN may affect both pathogenic and protective cells (103) or other tissue functions (314). AD metabolic dysregulation is systemic, involving interactions between tissue stromal cells (e.g., RA FLS) and distant organs (177, 178), adding complexity. Therefore, it is essential to move beyond one-size-fits-all suppression and toward tailored and personalized interventions. Cellular metabolic pathways are evolutionarily conserved and shared among nearly all cell types. This universality means metabolic interventions often have broad systemic effects. Translating basic findings into effective clinical therapies is challenging. Many drugs targeting core pathways (e.g., LXR agonists, ACC inhibitors) show efficacy preclinically but face limitations due to potential systemic side effects (e.g., hepatic steatosis with LXR agonists). Achieving targeted delivery to specific immune cells or tissues to improve efficacy and reduce off-target effects is a major bottleneck.

Future breakthroughs rely on multidisciplinary integration. Combining single-cell transcriptomics, proteomics, metabolomics, and lipidomics will enable mapping detailed metabolic landscapes of distinct immune cell subsets in disease states at unprecedented resolution. This will further aid in identifying disease-specific metabolic vulnerabilities, discovering novel targets, and stratifying patients for precision medicine trials based on their metabolic phenotypes. High-throughput lipidomics/metabolomics may yield biomarkers reflecting disease activity, predicting treatment response, or distinguishing patient subtypes, enabling early diagnosis, personalized therapy, and treatment monitoring. Developing novel drug delivery systems [e.g., based on liposomes, exosomes (311)] for targeted delivery of metabolic modulators to specific immune cells or inflamed tissues is key to overcoming off-target effects. Integrating metabolomic biomarkers with dietary (e.g., Omega-3 PUFAs, ketogenic diets) and gut microbiome (e.g., probiotics, FMT) interventions will help build multi-dimensional “metabolism-immune-microecology” regimens. Combining these agents with established immunotherapies promises synergistic effects. This includes pairing them with biologics (e.g., TNF-α or IL-17 inhibitors, B cell-depleting agents) or JAK inhibitors, which could lead to superior disease control, permit lower doses of individual drugs, and reduce treatment-related toxicity.

In summary, targeting lipid metabolism opens a promising new avenue for treating ADs. Future research will strive to deepen our understanding of this complex regulatory network and translate this knowledge into precise metabolic intervention strategies that truly improve patients’ quality of life. This will require relentless exploration by basic scientists, as well as close collaboration among clinicians, pharmacologists, and the biotechnology industry. Such efforts will propel this exciting field from bench to bedside, ultimately enabling effective control and personalized management of ADs.

## Supplementary material

Supplementary Figure 1 Schematic illustration of the regulatory network governing lipid metabolism. Fatty acids (FAs) enter cells via CD36-mediated lipid uptake. Subsequent intracellular signaling involves the PI3K/AKT pathway, which modulates activity of AMPK and mTOR1. AMPK functions as a central regulator: it influences peroxisome proliferator-activated receptor α (PPARα)-dependent transcriptional programs and modulates sterol regulatory element-binding protein 1 (SREBP1) activity. Transcription factors SREBP1 and SREBP2 translocate to the nucleus to drive expression of genes for fatty acid synthesis (e.g., fatty acid synthase, FASN; acetyl-CoA carboxylase, ACC) and cholesterol synthesis (e.g., 3-hydroxy-3-methylglutaryl-CoA reductase, HMGCR; low-density lipoprotein receptor, LDLR), respectively. PPARα promotes fatty acid oxidation (FAO) by facilitating acetyl-CoA transport into mitochondria via carnitine palmitoyltransferase 1 (CPT1); within mitochondria, β-oxidation, the tricarboxylic acid (TCA) cycle, and ATP production occur. Additionally, the liver X receptor (LXR) regulates cholesterol efflux via target genes such as ATP-binding cassette transporter A1 (ABCA1) and G1 (ABCG1).Created in BioRender. yu, Y (2025). https://BioRender.com/havvjba

Supplementary Table 1 Diverse lipids orchestrate immune cells in autoimmunity.
