# Supplementary figures and images for: Immunocyte lipid metabolic reprogramming: a novel pathway for targeted intervention in autoimmune diseases
Source: Front Immunol. 2025 Nov 6;16:1713148. doi: 10.3389/fimmu.2025.1713148 (PMC12631377; doi:10.3389/fimmu.2025.1713148)

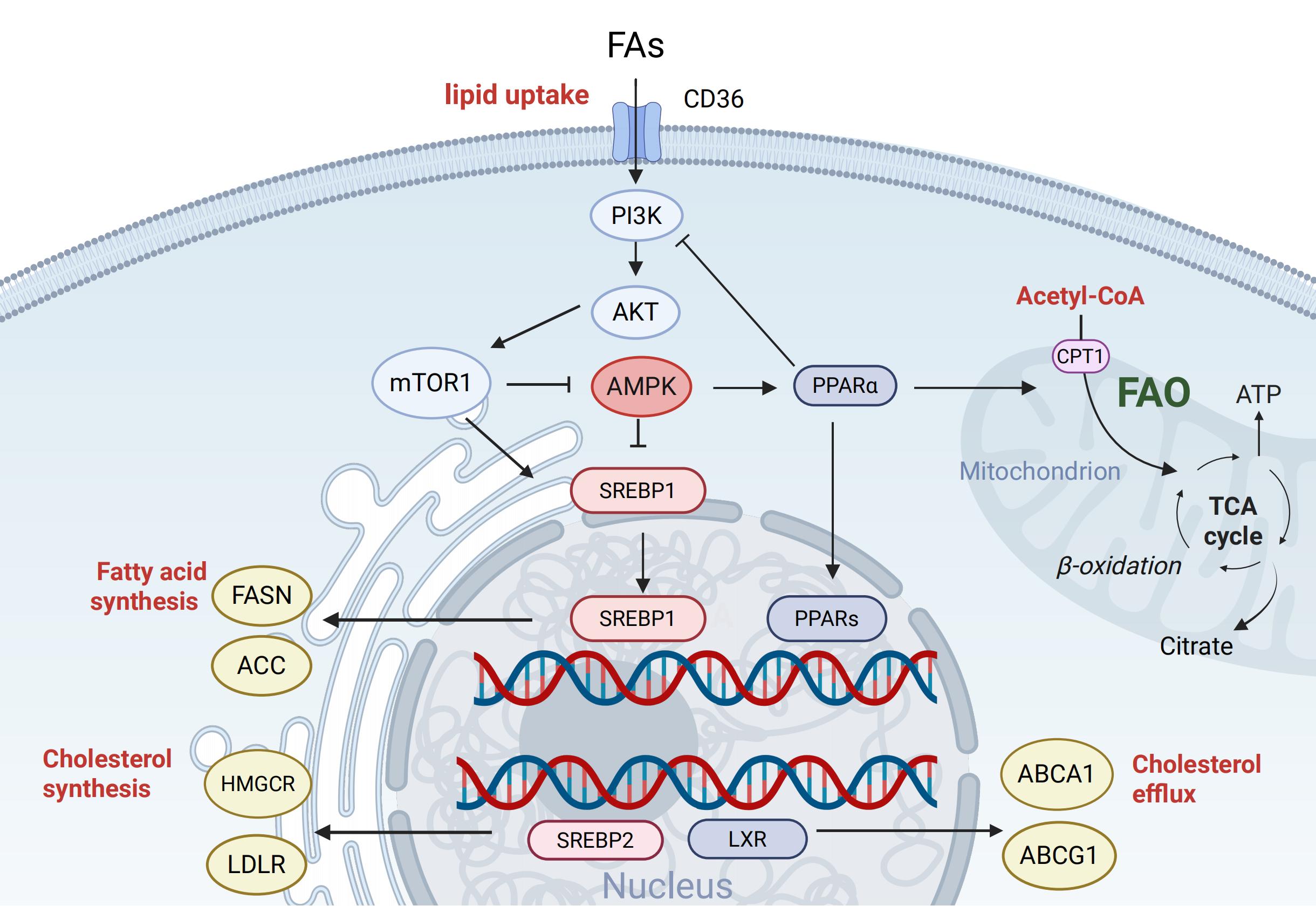

Supplement: Supplementary Figure 1 — Schematic illustration of the regulatory network governing lipid metabolism. Fatty acids (FAs) enter cells via CD36-mediated lipid uptake. Subsequent intracellular signaling involves the PI3K/AKT pathway, which modulates activity of AMPK and mTOR1. AMPK functions as a central regulator: it influences peroxisome proliferator-activated receptor α (PPARα)-dependent transcriptional programs and modulates sterol regulatory element-binding protein 1 (SREBP1) activity. Transcription factors SREBP1 and SREBP2 translocate to the nucleus to drive expression of genes for fatty acid synthesis (e.g., fatty acid synthase, FASN; acetyl-CoA carboxylase, ACC) and cholesterol synthesis (e.g., 3-hydroxy-3-methylglutaryl-CoA reductase, HMGCR; low-density lipoprotein receptor, LDLR), respectively. PPARα promotes fatty acid oxidation (FAO) by facilitating acetyl-CoA transport into mitochondria via carnitine palmitoyltransferase 1 (CPT1); within mitochondria, β-oxidation, the tricarboxylic acid (TCA) cycle, and ATP production occur. Additionally, the liver X receptor (LXR) regulates cholesterol efflux via target genes such as ATP-binding cassette transporter A1 (ABCA1) and G1 (ABCG1).Created in BioRender. yu, Y (2025). https://BioRender.com/havvjba [file Image1.jpeg]
