# Supplementary material for: Immunocyte lipid metabolic reprogramming: a novel pathway for targeted intervention in autoimmune diseases
Source: Front Immunol. 2025 Nov 6;16:1713148. doi: 10.3389/fimmu.2025.1713148 (PMC12631377; doi:10.3389/fimmu.2025.1713148)
Supplement: Supplementary Table 1 — Diverse lipids orchestrate immune cells in autoimmunity. [file Table2.docx]

**Table S1. Diverse Lipids Orchestrate Immune Cells in Autoimmunity**

| **Lipid Category** | **Specific Molecular Examples** | **Targeted Immune Cells** | **Pronflammatory/Anti-inflammatory** | **Role in Autoimmune Diseases** | **References** |
| --- | --- | --- | --- | --- | --- |
| Short-chain fatty acids | Acetate, Butyrate, Propionate | Treg, Th17 | ↓ | Promotes Treg function, alleviates inflammation in IBD and MS; inhibits Th17 differentiation | (308,309) |
| Long-chain fatty acids (Saturated) | Palmitate | Teff, Macrophages, γδ T17 cells | ↑ | Drives IL-17 production (e.g., by γδ T17); induces autoimmune B cell differentiation. | (86,310) |
| Long-chain fatty acids (Polyunsaturated, ω-6) | Arachidonic Acid | T cells, Macrophages, DCs | ↑ | Involved in pro-inflammatory responses in MS | (295,311) |
| Long-chain fatty acids (Polyunsaturated, ω-3) | DHA, EPA | T cells, Macrophages, DCs | ↓ | Inhibits pathogenic T(Th1/Th17) and B cells; alleviates lupus/EAE; modulates DCs via GPR120; ameliorates psoriatic inflammation. | (194,242,297,312) |
| Monounsaturated fatty acids | Oleate | Treg | ↓ | Promotes Treg differentiation, alleviates MS; regulates CD4+ T cell differentiation in Vogt-Koyanagi-Harada disease | (280,313-315) |
| Oxidized Lipids | Oxidized LDL (oxLDL) | B cells, Macrophages, DCs | ↑ | Pro-inflammatory via LOX-1/CD36; Exacerbates psoriasis, EAE, RA; CXCL16-dependent oxLDL clearance drives pathogenic CD8+ T cell differentiation. | (307,316) |
| Cholesterol | Cholesterol | T cells, B cells, Macrophages / Foam cells | Context-dependent | Enhances immune synapse & TCR signaling; Potentiates T/B cell responses; Elevates atherosclerosis risk in SLE. | (73,317) |
| Sphingolipids | Ceramide; S1P (Sphingosine-1-phosphate) | T cells, Macrophages / Microglia, DCs | Context-dependent | Ceramide promotes inflammation; S1P regulates lymphocyte migration, and its receptor-targeting drugs are used for MS and IBD treatment. | (214,318) |
| Phospholipids & Metabolites | Lysophosphatidylcholine (LPC) / Lysophosphatidic acid (LPA) | DCs, Macrophages, T cells, B cells | ↑ | LPA promotes neuroinflammation and inhibits remyelination; specific phospholipid metabolic pathways drive Th17 differentiation. | (168,276) |
| Bioactive Lipid Mediators (Eicosanoids) | PGE₂ (Prostaglandin E₂), LTB₄ (Leukotriene B₄) | Th17/Th1/Treg, γδ T17 cells, DCs | ↑↓ | PGE₂/LTB₄: exacerbate RA; LXA₄: resolves inflammation, modulates Th17/Treg, ameliorates EAE. | (233,294) |
| Lipid Droplets / Neutral Lipids (TGs, Cholesteryl Esters) | Triacylglycerols | Activated lymphocytes, Macrophages | ↑ | Provides energy for activated lymphocytes, promotes their migration and inflammatory effects, exacerbates RA and MS. | (147) |
